# PUTH Grading System for Urinary Tumor With Supradiaphragmatic Tumor Thrombus: Different Surgical Techniques for Different Tumor Characteristics

**DOI:** 10.3389/fonc.2021.735145

**Published:** 2022-01-06

**Authors:** Zhuo Liu, Yuxuan Li, Yu Zhang, Xun Zhao, Liyuan Ge, Shiying Tang, Peng Hong, Shudong Zhang, Xiaojun Tian, Shumin Wang, Cheng Liu, Hongxian Zhang, Lulin Ma

**Affiliations:** ^1^ Department of Urology, Peking University Third Hospital, Beijing, China; ^2^ Department of Ultrasound, Peking University Third Hospital, Beijing, China

**Keywords:** extracorporeal circulation, inferior vena cava, modified technique, urinary tumor, renal cell carcinoma, supradiaphragmatic tumor thrombus, transabdominal approach

## Abstract

**Purpose:**

To explore the different treatment strategies for urinary tumors with Mayo IV thrombus.

**Materials and Methods:**

We retrospectively analyzed the patients with Mayo IV thrombus in Peking University Third Hospital from January 2014 to April 2021. We used the Peking University Third Hospital (PUTH) grading system to classify urinary tumors with supradiaphragmatic thrombus. PUTH-A referred to the filled thrombus whose tip just reached above the diaphragm, or the thrombus entering the right atrium (< 2cm). PUTH-B referred to the filled thrombus entering the right atrium (> 2cm), or the thrombus invading the wall of the inferior pericardial vena cava. Detailed techniques were described for various scenarios. Clinicopathological data and perioperative outcomes were reported. Group difference statistical analysis was performed.

**Results:**

A total of 26 cases of urinary tumors with supradiaphragmatic IVC thrombus (Mayo grade IV) underwent treatment were enrolled in this study. 19 patients in the PUTH-A group received the open approach without sternotomy and cardiopulmonary bypass. Seven patients in the PUTH-B group received open thoracotomy assisted by cardiopulmonary bypass. No intraoperative death occurred. After 56 months of follow-up, 46.2% (12 of 26) patients died of all causes. Estimated 1-year, 2-year, and 3-year overall survival were 72.0% (95% CI, 54.4%-89.6%), 58.2% (95% CI, 38.0%-78.4%), and 52.4% (95% CI, 31.2%-73.6%), respectively.

**Conclusions:**

We introduced the PUTH grading system for the characteristics of urinary tumors with supradiaphragmatic tumor thrombus, and selected different surgical techniques according to different classifications. This grading system was relatively feasible and effective.

## Introduction

Renal tumors have the tendency to involve the venous system (1). In some patients the thrombus can invade the renal vein as well as the inferior vena cava (IVC) and even involve the right atrium (RA) (2–4). It was reported that 4% to 10% of the patients with renal cell carcinoma (RCC) had venous thrombus and 1% RCC patients had thrombus involving the RA (5, 6). The thrombus involving the RA was classified to be Mayo IV level and was thought to be tough to operate because of the complicated vascular control (7). Atrial incision under cardiopulmonary bypass and removal of intraatrial thrombus in a bloodless environment is the preferred treatment option (2, 3, 8). However, it can also result in some major complications such as dysfunction of coagulation (9, 10). For smaller tumor thrombus into the RA and no involvement of the pericardial wall or IVC wall, it can be operated by just opening the diaphragm without opening the chest (11, 12). The surgical options depend on the characteristics of thrombus and tumor. Accordingly, the purpose of this study was to explore the different treatment strategies for urinary tumors with Mayo IV thrombus.

## Materials and Methods

### Data Collection

Approved by the Medical Ethics Committee of our hospital, we retrospectively analyzed the patients with Mayo IV thrombus in Peking University Third Hospital from January 2014 to April 2021. The presence of local symptoms was defined as palpable mass, pain, gross hematuria. Patients with edema, fever, swelling, fatigue and weight loss, et al. were thought to have systemic symptoms. The American society of anesthesiologists (ASA) was used to assess patients’ anesthetic and surgical risks. Enhanced computed tomography (CT) of urinary tract was used to confirm the clinical diagnosis of retroperitoneal tumors and to determine the presence of hilar lymph node metastasis and distant metastasis. Enhanced magnetic resonance imaging (MRI) was used to identify the characteristics of tumor thrombus in IVC and to assess the presence of accompanied blood thrombus (non-neoplastic thrombus) (13). The size of the tumor thrombus in the IVC were measured in the coronal view. According to the imaging characteristics, whether the tumor thrombus invaded the vascular wall was judged (14). Chest CT examination was performed to determine whether there was pulmonary metastasis. For patients with bone pain and central nervous system symptoms, bone scan and head MRI were performed to evaluate bone metastasis and brain metastasis. When necessary, PETCT was performed to assess the systemic metastasis. All patients were treated with multi-disciplinary treatment (MDT). The MDT team included urology, anesthesia, radiology, ultrasound diagnosis, oncology, pathology, general surgery, and cardiac surgery. MDT clinical treatment decisions were carried out on the basis of comprehensive opinions of various disciplines, including preoperative preparation, surgical methods, coping strategies of intraoperative special conditions, and prevention and treatment strategies of postoperative complications.

**Figure 1:**
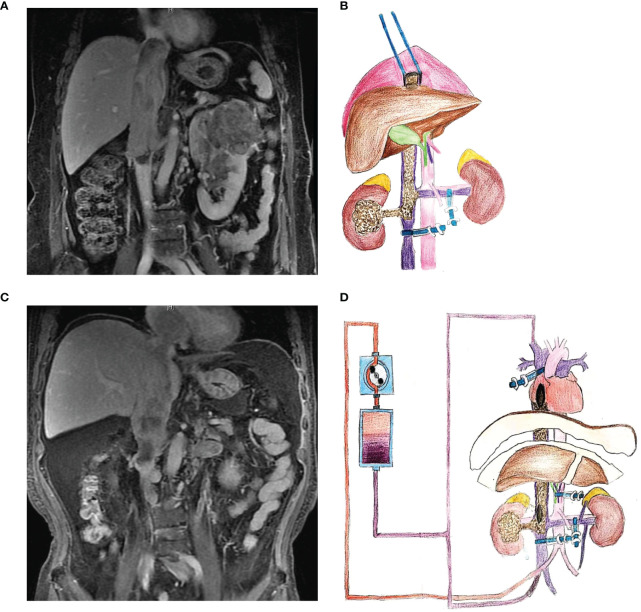
Preoperative imaging examination and surgical schematic diagram. **(A, B)** Imaging data and schematic diagram of open approach with incision of diaphragm without thoracotomy. **(C, D)** Imaging data and schematic diagram of cardiopulmonary bypass.

We used the Peking University Third Hospital (PUTH) grading system to classify urinary tumors with supradiaphragmatic thrombus. PUTH-A referred to the filled thrombus whose tip just reached above the diaphragm, or the thrombus entering the right atrium (< 2cm). The surgical approach for such thrombus was usually an open approach: incision of the diaphragm without thoracotomy. PUTH-B referred to the filled thrombus entering the right atrium (> 2cm), or the thrombus invading the wall of the inferior pericardial vena cava. The procedure for such thrombus was usually the open thoracoscopic therapy assisted by cardiopulmonary bypass. We summarized the surgical indications, position and incision, keypoint and special techniques of the different PUTH classifications in **Table 1**. The preoperative imaging characteristics and surgical schematic diagram of the two group patients were shown in **Figure 1**.

**Table 1:** Peking University Third Hospital (PUTH) grading system to classify urinary tumors with supradiaphragmatic thrombus.

Surgical Approach	Open Approach: Incision of Diaphragm Without Thoracotomy (n = 19)	Open Thoracotomy Assisted by Cardiopulmonary Bypass (n = 7)
Name	PUTH-A	PUTH-B
Indications	the filled thrombus whose tip just reached above the diaphragm, or the thrombus entering the right atrium (< 2cm)	the filled thrombus entering the right atrium (> 2cm), or the thrombus invading the wall of the inferior pericardial vena cava
Position	Supine position	Supine position
Incision	Chevron incision	Chevron incision, Midthoracic incision
The key steps of the surgical procedure	(A) Dissociate the liver and expose the posterior hepatic IVC (B) Dissociate the distal IVC (IVC under the renal vein), the contralateral renal vein and First portal hepatic vessel (C) Open the diaphragm and the pericardium. The “Milking” technique was used, in which the intraatrial thrombus was squeezed into the IVC and the upward passage of the thrombus was blocked. (D) Block the distal IVC, the contralateral renal vein, the first portal hepatic vessels and the proximal IVC (E) Remove the thrombus by balloon catheterization after incision of the IVC wall, and then suture the IVC wall. Move the IVC occlusion band above the thrombus to the lower liver and open the occlusion band in the first hilum to shorten the time of hepatic ischemia. (F) Remove the vascular occlusion bands after complete suturing of the IVC wall	(A) Open the pericardium to expose the heart and the main vessels, while separating the femoral arteries and veins in the inguinal area. After heparinization, the femoral artery, femoral vein and superior vena cava were intubated, followed by extracorporeal circulation. (B) Open the atrium and remove the thrombus in a bloodless environment. (C) Block the IVC at the superior diaphragmatic level, followed by occlusion of the distal IVC, the contralateral renal vein, and the first portal hepatic vessel. (D) Cut the IVC wall longitudinally at the point where the renal vein entered. Remove the thrombus in the abdominal IVC by balloon catheter technique and then remove the kidney (E) Suture the atrium (F) Gradually stop cardiopulmonary bypass and give protamine to reverse the effect of heparin. (G) Suture the IVC continuously (H) Loosen the occlusion bands of the proximal IVC, the first portal hepatic vessels, the contralateral renal vein, and the distal IVC successively.
Special techniques	Balloon catheterization technique	Balloon catheterization technique
Dissociate the liver	Yes	Yes
Block the first porta vessels	Yes	Yes
The blood vessels blocked	Distal IVC, contralateral renal vein, proximal IVC and first porta vessels	Distal IVC, contralateral renal vein, proximal IVC and first porta vessels

Postoperative complications were assessed using a modified Clavien grading system (15, 16). Complications above III grade were defined as serious complications (17). The first follow-up was conducted within 1 month after surgery, with a focus on postoperative complications. Follow-up was then conducted every 3-6 months in the first year, every 6 to 12 months in the second year and then annually after surgery.

### Surgical Technique

#### Technique of Incision of Diaphragm Without Thoracotomy

Chevron incision was made and peritoneum was incised along the Toldt line. The hepatic curvature of the colon and the duodenum were pushed medially. The renal artery and vein were dissociated successively, and then the renal artery was ligated and cut off. The affected ureter was dissociated and ligated and cut off. The left renal artery and vein were isolated and cupped for occlusion. We dissociated the IVC and ligate the lumbar vein and other vein branches to the IVC. The round ligament and falcate ligament of liver were severed and the liver was dissociated from the diaphragm and pulled down to expose the coronal ligament of liver. We cut off the left and right hepatic deltoid ligaments and then open the retroperitoneal fold between the liver and the right kidney and release the right lobe of the liver. The liver was rotated medially to expose and dissociate the posterior and superior hepatic IVC. It was also feasible to perform piggyback liver dissociation: after cutting off the left and right hepatic triangle ligaments, the liver was gradually turned over and dissociated to the left, the right supra coronal ligament was exposed and cut off, the liver was further turned over to the left, and the short hepatic vein from the right hepatic lobe and caudate lobe to the posterior hepatic IVC was cut off, and the posterior and superior hepatic IVC was dissociated. The central diaphragmatic tendon was dissociated, and part of the diaphragmatic IVC was pulled down or cut open to expose the superior diaphragmatic IVC to the RA. The diaphragm was opened lengthwise and the pericardium was incised to expose the heart. The “Milking” technique was used, in which the intraatrial thrombus was squeezed into the IVC by finger compression and the upward passage of the tumor thrombus was blocked. The IVC cuff was placed over the thrombus for occlusion. The exposed hepatic portal vessels were isolated and cuffed for occlusion. Successively blocking the distal IVC, the contralateral renal vein, hepatic portal vessels and the proximal IVC. We then cut the IVC and put into the balloon urinary canal cross the upper thrombus. After that we removed the thrombus completely and performed resection of kidney. If the thrombus was adhered to the IVC wall, we sharp freed the thrombus. When necessary, we removed IVC wall or perform segmental resection of IVC. After that, we closed the IVC incision. The occlusion forceps above the thrombus were moved to the subhepatic IVC and the first portal was opened to shorten the occlusion time of liver. After washing the IVC to confirm that there was no residual tumor thrombus, we sutured the IVC completely. Then, the subhepatic IVC, the contralateral renal vein and the distal IVC were opened successively. After washing the wound and stopping the bleeding sufficiently, the incision was closed layer by layer.

#### Open Thoracotomy Assisted by Cardiopulmonary Bypass

The Chevron incision was extended to xiphisternal horn or the thoraco-abdominal midline incision was made. We opened the pericardium to expose the heart and the large blood vessels, while the femoral arteries and veins were separated in the inguinal area. After heparinization, the ascending aorta, femoral vein and superior vena cava (SVC) were intubated, and extracorporeal circulation was initiated. According to the situation we chose deep hypothermic circulation arrest (DHCA) or no circulation arrest with hypothermic low flow technology. We opened the RA and removed the thrombus in the RA in a bloodless environment. The IVC was occluded at the diaphragmatic level, followed by the distal IVC, the contralateral renal vein, and the first portal hepatic vessel. The IVC wall was cut longitudinally at the point where the renal vein entered. The thrombus in the abdominal IVC was removed by balloon catheterization and the kidney was resected. The IVC was flushed with heparin saline. Then, from the RA downwards and the incision of the IVC upwards, the RA was continuously sutured after finger examination to confirm no residual thrombus. Cardiopulmonary bypass was gradually stopped and protamine was given to reverse the effect of heparin. Mediastinal pleural drainage tubes were routinely placed. If coagulation disorders occurred, platelets, plasma, vasopressin were given. The IVC was sutured continuously, and the proximal IVC, the first hepatic portal occlusion band, the contralateral renal vein and the distal IVC occlusion band were successively loosened.

### Statistical Analysis

A normal test was performed for continuous variables. For the normal distribution, the mean ± standard deviation was used. The clinical and pathological features of the two groups were analyzed using Independent Samples T-test. For non-normal distribution data, continuous variables were described as medians and interquartile ranges (IQRs), and non-parametric tests were used to compare whether there were differences between the two groups of distributions. The categorical variables were described as numbers and proportions, and the difference of proportion between groups was compared by the chi-square test. Those who did not meet the chi-square test were compared by Fisher’s exact probability method. P<0.05 was considered statistically significant.

## Results

A total of 26 patients with surgical treatment of urinary tumors with supradiaphragmatic IVC thrombus (Mayo grade IV) were contained in this analysis. The average age is 60.7 years old and the average BMI is 24.0kg/m^2^. There were 21(80.8%) males and 5(19.2%) females. There were 18 cases of retroperitoneal tumors on the right and 8 cases on the left side. According to the American society of anesthesiologists (ASA) score, there were 15 cases of grade II, 10 cases of grade III and one case of grade IV.

Nineteen patients received the open approach without sternotomy and cardiopulmonary bypass. Seven patients received open thoracotomy assisted by cardiopulmonary bypass. No intraoperative death occurred. The operative time of the two groups were 400.1 ± 87.9min and 564.9 ± 80.5min, respectively. The operative time of PUTH A group was significantly shorter (p<0.001). There was no significant difference in intraoperative surgical blood loss (p=0.067) between the two groups, and there was no significant difference in the incidence of overall complications and severe complications between the two groups, either.

Complications occurred in 12 of 19 (63.2%) patients in the PUTH A group. One case had wound infection (Clavien Grade I) and recovered after dressing change. Five cases had Clavien grade II complications, which were venous thrombosis of lower limbs (1 case), anemia (1 case), anemia (1 case), thrombocytopenia (1 case), thrombocytopenia (1 case), incomplete intestinal obstruction (1 case) and mild decrease of liver function (1 case). Severe complications occurred in 6 patients (31.6%), including 4 patients with Clavien grade IV. Among them, 3 patients with renal failure improved after hemodialysis and 1 patient with multiple organ dysfunction recovered after symptomatic anti-infection supportive treatment. Two patients with Clavien grade V died of multiple organ failure 2 and 4 days after surgery.

Complications occurred in 4 of 7 (57.1%) patients in PUTH B group. Two cases had Clavien Grade II complications including 1 case of anemia which recovered after blood transfusion and 1 case of pulmonary infection which improved after anti-infection treatment. Severe complications occurred in 2 patients (28.6%), of which 2 patients were classified as Clavien V. One patient suffered from blood pressure drop and died of cardiac arrest 1 day after surgery, and another patient suffering from septic shock, multiple organ dysfunction and coagulation insufficiency died 14 days after surgery. Only 2 PUTH-A patients received neoadjuvant therapy, 10 of 19 (52.6%) PUTH-A and 4 of 7 (57.1%) PUTH-B patients received adjuvant therapy. There was no significant difference between the two groups (p=1.000). Detailed clinicopathologic characteristics are described in **Table 2**.

**Table 2:** Clinicopathologic characteristics of the two groups patients.

Surgical Approach	Open Approach: Incision of Diaphragm Without Thoracotomy (n = 19)	Open Thoracotomy Assisted by Cardiopulmonary Bypass (n = 7)	p
Gender, n (%)			0.278
Male	14 (73.7)	7 (100)	
Female	5 (26.3)	0 (0)	
Age, y, mean ± SD	61.1 ± 9.3	59.4 ± 6.0	0.663
BMI, kg/m^2^, mean ± SD	24.5 ± 4.3	22.7 ± 1.6	0.296
Side, n (%)			0.635
Left	5 (26.3)	3 (42.9)	
Right	14 (73.7)	4 (57.1)	
ASA grade, n (%)			0.546
2	12 (63.2)	3 (42.9)	
3	6 (31.6)	4 (57.1)	
4	1 (5.3)	0 (0)	
Clinical symptoms, n (%)			0.017*
No clinical symptoms	4 (21.1)	0 (0)	
Local symptoms	9 (47.4)	3 (42.9)	
Systemic symptoms	5 (26.3)	0 (0)	
Both	1 (5.3)	4 (57.1)	
Clinical N stage, n (%)			0.178
cN0	10 (52.6)	1 (14.3)	
cN1	9 (47.4)	6 (85.7)	
Clinical M stage, n (%)			1.000
cM0	12 (63.2)	4 (57.1)	
cM1	7 (36.8)	3 (42.9)	
Tumor diameter, cm, mean ± SD	8.2 ± 2.2	7.4 ± 2.4	0.421
Presence of bland thrombus, n (%)	10 (52.6)	2 (28.6)	0.391
Branch tumor thrombus, n (%)	6 (31.6)	3 (42.9)	0.661
IVC thrombus length, cm, mean ± SD	14.4 ± 3.5	13.4 ± 2.3	0.511
IVC thrombus width, cm, mean ± SD	3.5 ± 0.8	3.1 ± 0.9	0.309
IVC transverse resection, n (%)	6 (31.6)	2 (28.6)	1.000
Ipsilateral adrenalectomy, n (%)	9 (47.4)	5 (71.4)	0.391
Operative time, min, mean ± SD	400.1 ± 87.9	564.9 ± 80.5	<0.001*
Surgical blood loss, mL, mean ± SD	2330.0 ± 1862.1	4200.0 ± 3004.4	0.067
Pathological type, n (%)			0.127
Clear cell RCC	17 (89.5)	5 (71.4)	
Papillary RCC	0 (0)	2 (28.6)	
Chromophobe RCC	1 (5.3)	0 (0)	
Unclassified RCC	1 (5.3)	0 (0)	
Sarcomatoid differentiation, n (%)	4 (21.1)	0 (0)	0.546
Pathological renal hilar lymph node metastasis, n (%)	1 (5.3)	2 (28.6)	0.167
Preoperative serum creatinine, µmol/L, mean ± SD	84.1 ± 17.7	104.4 ± 18.8	0.017*
Serum creatinine one week after operation, µmol/L, median (IQR)	93.0 (74.0,196.0)	95.0 (86.0,103.0)	0.778
Postoperative hospital stay, d, median (IQR)	10.0 (8.0,21.0)	12.0 (9.0,14.0)	0.955
Postoperative complication, n (%)	12 (63.2)	4 (57.1)	1.000
Severe complication, n (%)	6 (31.6)	2 (28.6)	1.000
Neoadjuvant therapy, n (%)	2 (10.5)	0 (0)	1.000
Adjuvant therapy, n (%)	10 (52.6)	4 (57.1)	1.000

*p < 0.05.

**Figure 2:**
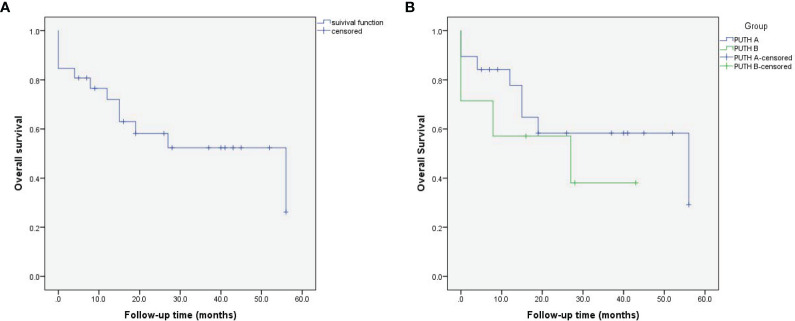
Kaplan-Meier curves for overall survival in patients with supradiaphragmatic IVC thrombus (Mayo grade IV) (log rank test p=0.407). **(A)** All patients, **(B)** PUTH A, B group.

All the 26 patients were followed up and after 56 months of follow-up, 46.2% (12 of 26) patients died of all causes. Estimated 1-year, 2-year, and 3-year overall survival were 72.0% (95% CI, 54.4%-89.6%), 58.2% (95% CI, 38.0%-78.4%), and 52.4% (95% CI, 31.2%-73.6%), respectively. (**Figure 2A**). There was no statistically significant difference between the PUTH-A group and PUTH-B group (p=0.407, **Figure 2B**).

## Discussion

The clinical manifestations of renal carcinoma patients with Mayo-IV grade thrombus include local symptoms such as hematuria, low back pain and abdominal mass, as well as systemic symptoms such as fatigue, weight loss, anemia and hypertension. A few patients presented symptoms of IVC obstruction such as lower extremity edema, varicocele and superficial abdominal varicose veins and intraatrial thrombus related symptoms such as chest dampness and shortness of breath, night sweats and dizziness (18–20). It has been shown that about 95% of such patients had symptoms (21). In our study cohort, 22 patients (84.6%) developed clinical symptoms.

Preoperative preparation is more important for such patients and it need multidisciplinary cooperation including hepatobiliary surgery, anesthesiology, cardiothoracic surgery, vascular surgery and other departments to discuss and develop the surgical plan together. For patients with atrial tumor thrombus, preoperative chest CT and echocardiography were needed to determine the burden of thrombus and its influence on the right heart. About 5% of patients with grade Mayo-IV thrombus had preoperative pulmonary embolism (PE) (18). One patient had preoperative pulmonary embolism in our group and died perioperatively. The relationship between preoperative PE and prognosis still needs further study. By transesophageal echocardiography (TEE), we can detect the structure, function and capacity of the heart in real time and evaluate the location of thrombus. TEE has advantages including detection of thrombus shedding, detection of right ventricular outflow obstruction, PE and other serious complications and helping physician evaluate surgical effect. All patients in this group were monitored by TEE, and no tumor thrombus was found to fall off.

Urinary tumor with IVC thrombus is one of the most difficult urological operations. The surgical option depends largely on the location of the upper pole of the thrombus. Supradiaphragmatic thrombus can be classified as grade IV according to the Mayo grading system and as grade IIId if the thrombus didn’t reach right atrium according to the Ciancio grading system (12). The difficulty of supradiaphragmatic thrombus lies in the treatment of the proximal end of thrombus.

In this paper, we classified urinary tumors with supradiaphragmatic thrombus and introduced this grading system, PUTH classification. Different classifications need different surgical treatment. The traditional surgical approach for supradiaphragmatic thrombus is to perform extracorporeal circulation after thoracotomy. Hypothermic cardiac arrest and atrial incision could be used to remove the tumor thrombus which is to obtain a clear view for thrombectomy. If the thrombus fall off during operation, this method can help avoid pulmonary embolism effectively. This surgical technique is mainly applicable to the long entry segment of thrombus into atrium(>2cm) or the thrombus invade the inferior pericardial vena cava wall, which causes the thrombus to be unable to push. The surgical approach of cardiopulmonary bypass (CPB) with open chest may lead to the relative complications, such as coagulation dysfunction, acute renal insufficiency, sepsis, and multiple organ dysfunction. For thrombectomy with hypothermic cardiac arrest, the perioperative mortality reaches 3% to 16% (12). Although some scholars believe that CPB does not increase the survival rate of supradiaphragmatic thrombectomy (22), we still need to pay high attention to the complications caused by CPB.

The technique of opening the diaphragm without opening the chest described in this paper can effectively avoid CPB. The key point of the technique is opening the central tendon of the diaphragm to expose the entry of the superior vena cava into the atrium. The Milking technique is to press the thrombus in the atrium into the IVC and block the upward migration pathway of the thrombus, then block the IVC at the proximal thrombus. The advantage of technique is that it can effectively avoid complications associated with CPB. However, the disadvantage is that the length of the thrombus into the atrium is required. This technique is mostly applied to patients with thrombus just reaching above the diaphragm level or within 2cm of the atrium. For patients with thrombus extending into the atrium longer than 2cm, CPB technique is mostly used.

The perioperative mortality of Mayo-IV grade thrombus is as high as 10% ~ 40% (21, 23). The main causes are cardiac arrest, tumor embolism and haemorrhage, sepsis and multiple organ failure caused by hemodynamic disorders. The incidence of intraoperative PE was 1.5% and the mortality was 75% (24). The perioperative mortality of this group of patients was 15.4%, consistent with other reports. The results showed that the survival of patients with renal carcinoma with Mayo-IV grade thrombus was short, and the 1-year disease-specific survival (DSS) of patients without distant metastasis was short. The DSS rate was 41% and the median DSS was 8 months for patients with M0 disease. The DSS rate was 10% and the median DSS was 3 months in patients with distant metastasis (25). Active surgery can significantly improve the patient’s prognosis. The 5-year tumor-specific survival rate was 36.1%, and the median survival was 19.3 months (26).

We acknowledge that there are some limitations in this study. Although the data comes from a large sample center in China, the data is provided by one single center. Uncontrolled confounding factors and inherent selection bias may exist. In addition, the number of patients is limited, although it is a study with a large number of cases reported at present, multi-center and larger sample studies can be carried out. Moreover, although we tried to follow up with all the patients until June 2021, the follow-up time was still relatively short. We need long-term follow-up to improve our results.

## Conclusions

We introduced the PUTH grading system for the characteristics of urinary tumors with supradiaphragmatic tumor thrombus, and selected different surgical techniques according to different classifications. This grading system was relatively feasible and effective.

## Data Availability Statement

The raw data supporting the conclusions of this article will be made available by the authors, without undue reservation.

## Ethics Statement

The studies involving human participants were reviewed and approved by Peking University Third Hospital ethics committee. The patients/participants provided their written informed consent to participate in this study. Written informed consent was obtained from the individual(s) for the publication of any potentially identifiable images or data included in this article.

## Author Contributions

ZL, YL, YZ, HZ, and LM: study conception and design, clinical studies, literature search, statistical analysis, data analysis, manuscript preparation, manuscript editing. XZ, LG, ST, and PH: study conception and design, clinical studies, literature search, data analysis, manuscript editing. SZ, XT, SW, and CL: study conception and design, clinical studies, manuscript editing. HZ and LM: guarantor of the integrity of the entire study. The authors have read and approved this manuscript, and ensure that the listed authors’ contributions are accurate.

## Funding

This study was supported by the National Nature Science Foundation of China (NO. 81771842), National Nature Science Foundation of China (NO. 82072211).

## Conflict of Interest

The authors declare that the research was conducted in the absence of any commercial or financial relationships that could be construed as a potential conflict of interest.

## Publisher’s Note

All claims expressed in this article are solely those of the authors and do not necessarily represent those of their affiliated organizations, or those of the publisher, the editors and the reviewers. Any product that may be evaluated in this article, or claim that may be made by its manufacturer, is not guaranteed or endorsed by the publisher.
